# Partnering With Mommy Bloggers to Disseminate Breast Cancer Risk Information: Social Media Intervention

**DOI:** 10.2196/12441

**Published:** 2019-03-07

**Authors:** Kevin Wright, Carla Fisher, Camella Rising, Amelia Burke-Garcia, Dasha Afanaseva, Xiaomei Cai

**Affiliations:** 1 George Mason University Fairfax, VA United States; 2 University of Florida UF Health Cancer Center Gainesville, FL United States; 3 Westat Rockville, MD United States

**Keywords:** breast cancer, environment and public health, risk reduction behavior, blogging, social networking, social media, health communication, information dissemination

## Abstract

**Background:**

Women are concerned about reducing their breast cancer risk, particularly if they have daughters. Social media platforms, such as blogs written by mothers, are increasingly being recognized as a channel that women use to make personal and family health–related decisions. Government initiatives (eg, Interagency Breast Cancer and Environmental Research Coordinating Committee) and researchers have called for scientists and the community to partner and disseminate scientifically and community-informed environmental risk information.

**Objective:**

We developed and evaluated a blog intervention to disseminate breast cancer and environmental risk information to mothers. We teamed with *mommy bloggers* to disseminate a message that we developed and tailored for mothers and daughters based on scientific evidence from the Breast Cancer and the Environment Research Program (BCERP). We posited that the intervention would influence women’s exposure to, acceptance of, and beliefs about environmental risks while promoting their intention to adopt risk-reducing behaviors.

**Methods:**

Using a quasi-experimental design, we recruited 75 mommy bloggers to disseminate the breast cancer risk message on their respective blogs and examined the impact of the intervention on (1) readers exposed to the intervention (n=445) and (2) readers not exposed to the intervention (comparison group; n=353).

**Results:**

Following the intervention, blog reader scores indicating exposure to the breast cancer risk and prevention information were greater than scores of blog readers who were not exposed (or did not recall seeing the message; mean 3.92, SD 0.85 and mean 3.45, SD 0.92, respectively; *P*<.001). Readers who recalled the intervention messages also had higher breast cancer risk and prevention information satisfaction scores compared with readers who did not see (or recall) the messages (mean 3.97, SD 0.75 and mean 3.57, SD 0.94, respectively; *P*<.001). Blog readers who recalled seeing the intervention messages were significantly more likely to share the breast cancer risk and prevention information they read, with their daughters specifically, than readers who did not recall seeing them (χ^2^_1_=8.1; *P*=.004). Those who recalled seeing the intervention messages reported significantly higher breast cancer risk and prevention information influence scores, indicative of behavioral intentions, than participants who did not recall seeing them (mean 11.22, SD 2.93 and mean 10.14, SD 3.24, respectively; *P*=.003). Most women ranked Facebook as their first choice for receiving breast cancer risk information.

**Conclusions:**

Results indicated that blog readers who were exposed to (and specifically recalled) the BCERP-adapted intervention messages from mommy bloggers had higher breast cancer risk and prevention information exposure scores and higher breast cancer risk and prevention information satisfaction and influence scores than those who did not see (or recall) them. Mommy bloggers may be important opinion leaders for some women and key to enhancing the messaging, delivery, and impact of environmental breast cancer risk information on mothers.

## Introduction

### Overview

Breast cancer is the second most common cancer diagnosed in women [1]. Women, particularly those with a personal or family history of the disease and mothers with daughters, are concerned about what they can do to reduce the risk of breast cancer [2-7]. In addition, when mothers advise their daughters about how to reduce the risk, daughters tend to follow their advice into adulthood [7]. Although clinicians, scientists, and advocacy groups prioritize cancer screening to reduce the risk (eg, mammograms and genetic testing), scientists have discovered that environmental exposures (eg, having contact with certain chemicals or particles through food, air, water, or touch) impact women’s risk [8].

In response to increasing research on breast cancer risk and environmental exposures, Congress passed the *Breast Cancer and Environmental Research Act* in 2008. This resulted in the initiation of the Interagency Breast Cancer and Environmental Research Coordinating Committee (IBCERCC), which was tasked with examining the state of this research. Their report identified 7 recommendations, 2 of which centered on the need for scientists to become engaged in dissemination, specifically prioritizing community stakeholder engagement and translating research for the public [9].

The authors are members of 1 group actively involved in the creation and dissemination of such research—the Breast Cancer and the Environment Research Program (BCERP). Founded in 2003 and funded by the National Institute of Environmental Health Sciences (NIEHS) and the National Cancer Institute (NCI), BCERP researchers have conducted decades of studies linking environmental factors and breast cancer risk. This includes identifying endocrine-disrupting chemicals (eg, phthalates and bisphenol A [BPA]) that could increase women’s risk and are found in some personal care and household products (eg, shampoo, detergent, and plastic bottles) [8]. BCERP provides actionable steps that women and their family members can take to reduce their exposure to these environmental risks. In recent years, BCERP has funded projects focused specifically on dissemination. The program currently disseminates this evidence-based information free to the public via their website [10].

Although women seek Web-based information about how to reduce personal and familial risk of breast cancer [11], they may not be obtaining reputable or scientifically based information that organizations such as BCERP provide. Readability and accessibility are also issues. A recent review of breast cancer and environmental risk information online showed that most content is not disseminated at a readable level with health literacy in mind [12]. There is also a plethora of information on the internet from divergent sources, including medical websites that are not regulated [13]. Websites are discovered based on users’ search terms with no guarantee that reputable sources such as BCERP are even reached. Thus, as the IBCERCC report also identified, researchers need to do a better job of disseminating breast cancer information in ways that reach women. Dissemination approaches should also strive to reach more women or have the potential for rapid diffusion of information, the process by which information is spread across channels rapidly [14].

Social media offers an ideal dissemination channel with the potential to reach an expansive group of women. Current evidence [15] has shown that:

Social media are becoming preferred methods of health promotion as evidence builds showing their effectiveness in reaching public audiences...Evidence about social media’s impact on health knowledge, behavior, and outcomes shows these tools can be effective in meeting individual and population health needs.

Social media platforms, such as blogs, offer a way to disseminate health information rapidly and expansively, educate the public, and promote healthy behavior. Still, *careful application* and evaluation is needed to obtain the desired outcomes [15] as blog-based interventions can result in limited exposure, messages not being tailored to the targeted audience, or messages that do not account for health literacy or culture [12,13,16]. Also, when it comes to online dissemination about environmental breast cancer risk, accuracy is of concern [12]. This is particularly true with blogs [17], which have been shown to be the least accurate in comparison with other online sources [12].

A recent review also showed that online information about environmental exposures is particularly complicated and, at times, misunderstood [12], with more commercial-focused online sources reporting known risk associations within exposures like deodorant, which opposes research showing no associated risk presented on more credible sites (eg, American Cancer Society). Additionally, this review found that while research may be presented accurately, the conclusions are not always accurately drawn and appear to be driven by personal motivations [12].

To ensure access and accuracy in the dissemination of breast cancer risk information, scholars have recently called for a community-engaged approach, linking scientists with community stakeholders to ensure that the public has access to the information and that it is disseminated in a manner that is readable and relatable [18]. In line with the IBCERCC’s recommendation [9], mothers who blog, or *mommy bloggers*, are an ideal community partner to disseminate information via blogs to reach women. Their blog posts could integrate scientifically based information in a manner that blog readers can identify. Also, blogs written by mothers are increasingly recognized as a channel that women utilize to make personal and family health–related decisions [19-21], and as such, mommy bloggers are often viewed as relatable and trustworthy by other mothers.

In addition, evidence-based strategies are integral to successfully disseminating health information via blogs [15]. These strategies include tailoring messages with the audience or readers in mind. Tailoring may involve user-generated (ie, blogger-generated) content to promote acceptance (by using a trusted source that content readers identify with), encouraging multipronged approaches (multiple social media platforms), integrating theory during intervention development, and using tools to evaluate the impact of the intervention on readers [15,22].

With these evidence-based strategies in mind, we aimed to develop and evaluate a targeted blog intervention, teaming with mommy bloggers to disseminate scientifically informed breast cancer and environmental risk information (adapted from BCERP) with the potential for rapid diffusion. The goal was to influence readers’ (ie, mothers’) exposure to, acceptance of, and beliefs about environmental risks while promoting intentions to adopt risk-reducing behaviors. In addition to testing the efficacy of this approach, we sought to shed light on how such an intervention may extend farther than targeted readers or how readers may share the information across other platforms.

### Getting the Message Out There: Blogging About Reducing Breast Cancer Risk

For many, the internet is a primary source of health information, including information about reducing breast cancer risk [11,23,24]. People increasingly seek health information through social media, such as Facebook, Twitter, and blogs, where knowledge is shared within a relational network facilitated through an online community [25]. The interactive and relational nature of social media allows individuals to connect with and reach broader audiences to address a range of health issues [26,27]. Therefore, blogs may represent an optimal social media channel for disseminating breast cancer risk information [19].

Blogs can be rich in information and promote interaction among bloggers and readers. For instance, bloggers provide unique content for their readers compared with other online communities [28]. They display a keen understanding of readers’ health needs and beliefs [19,28] and typically deliver information in varied forms (eg, text, multimedia, and links to Web resources) that are shared interactively via bloggers’ posts and exchanges of readers’ comments [19,29]. This online interaction generates a larger community network, extending beyond the blog [30-32]. Bloggers and readers may share blog content online (eg, via Facebook, Twitter, and Pinterest) and offline (eg, with family, friends, coworkers, and community groups). Thus, blogging can reach a broad audience.

In addition to facilitating interaction and reach, blogs represent a unique channel where specific audiences (such as mothers) can be reached and influenced. Bloggers can customize health information in ways that readers relate to, as bloggers are likely perceived by readers as being similar in terms of beliefs, experiences, and language [33,34]. In other words, blog readers identify with the bloggers (eg, as a woman, mother, or survivor). This increases the likelihood of readers’ acceptance, sharing, and adoption of the information posted by the blogger [35,36].

Currently, there are about 3.9 million mothers in the United States who identify as a blogger, and many mothers with children at home turn to blogs for advice about health issues [19,37]. Blogs written by mothers or those that focus on motherhood topics—sometimes called *mommy blogs* —offer the possibility of reaching an important target audience for breast cancer risk information: women and their family members [20,38,39].

### Teaming With Mommy Bloggers for Rapid Diffusion of Information: Trusted Sources Women Can Identify With

Readers relate to their blogs in part because mommy bloggers routinely tailor messages to their target audience (eg, using strategies such as feminine rhetoric, humor, or personal stories with photographs that women identify with) [40-43]. Bloggers also direct their readers to relevant and accurate health information on the internet [44]. Given their trusted status among mothers, mommy bloggers could be used to disseminate risk-reducing information and persuade readers to adopt or respond to it [45]. In line with Rogers’ widely used diffusion of innovation theory [14], mommy bloggers may be key to disseminating such information.

### Influencing Mothers’ Risk-Reducing Perceptions and Behavior: Diffusion of Innovation Approach

As diffusion of innovation theory purports, early adopters or innovators (readers) and opinion leaders (bloggers) are central to the dissemination of new ideas to the public. Mommy bloggers could promote rapid diffusion of the information through larger blogging networks when bloggers adopt the message (ie, post the content and endorse it), which can influence readers to do the same. Thus, bloggers can be viewed as opinion leaders who may drive diffusion and uptake of environmental breast cancer risk information by their readers [14,19,45]. The risk-related content may heighten the urgency of the message, and the message’s effects may be further amplified through bloggers’ and readers’ comments, shares, and likes [46].

This interactive and dynamic diffusion of information also has the potential to influence women’s behavior, which is essential to reducing cancer risk. Blog intervention studies show that blogger-reader interactions can create a sense of immediacy about health topics that encourages readers’ adoption of healthy behaviors [47]. Moreover, the tailored context of the blog, in conjunction with readers’ perceived source similarity [35], can influence women’s adoption of recommended health behaviors [47].

Despite the potential for mommy bloggers to serve as trusted or relatable sources to disseminate breast cancer risk information to mothers, it is critical that bloggers disseminate accurate, scientifically informed content [12]. Moreover, content should in part be *user-generated* with a target audience in mind, meaning that it should be produced in part with the bloggers’ input [15]. To achieve this, collaborative message development (between scientists and bloggers) is warranted to ensure women receive accurate information in a format they trust and relate to.

### Targeting Mommy Blog Readers: Collaborative Message Development

As described, federally funded BCERP scientists and community partners have developed and disseminated research to bring awareness to the public about linkages between environmental exposures and breast cancer risk. BCERP provides several educational materials online, including a toolkit for mothers with daughters. These mother-daughter–focused materials specifically address how mothers can reduce their own and their daughter’s risk by adopting healthier lifestyle choices together (eg, eliminating products with BPA and phthalates).

Many mothers with daughters are concerned and feel uncertain about breast cancer prevention and their daughters’ risk [2,3,48]. However, when mothers advise daughters about how to reduce risk, daughters adhere to their mother’s advice into adulthood [7]. Talking about risk can be challenging. Younger daughters often avoid or withdraw from such conversations [4,49], which can trigger a physiological stress response[50]. Mothers have reported using third-party approaches (eg, a magazine article) to prompt interaction and ease their daughter’s comfort during discussions [3,4]. Relatedly, recent research shows that third-party Web-based approaches (eg, videos about BPA/perfluorooctanoic acid and radiation risk) favorably influence mothers’ and daughters’ prevention behavior [16,51].

Mommy bloggers’ posts could change mothers’ knowledge and beliefs about environmental breast cancer risk factors and simultaneously function as a third-party approach to facilitate mother-daughter communication. Even though the information on BCERP’s website is scientifically informed and expansive, the format and manner in which these materials (a brochure, flyer, and public service announcement) are delivered assume women will obtain them, first by finding their website and then by reading them on their own. Passive dissemination approaches such as these do not ensure the information reaches the target audience and, when used alone, are not likely to be translated to practice or result in behavioral change [52]. Moreover, these materials are often lengthy and not in a format ideal for social media dissemination.

A more active diffusion of information or dissemination approach on an interactive Web platform in which an influencer (eg, blogger) communicates with women (their readers) is more likely to reach the targeted audience. Additionally, this approach may prompt interaction within and outside the blog network. By teaming with mommy bloggers, the evidence-based information could be integrated into a user-generated format ideal for social media and delivered in a manner that their readers (mothers) can relate to.

### Research Foci

As the aforementioned research demonstrates, mothers look to mommy bloggers for health information. By partnering with mommy bloggers to disseminate evidence-based environmental risk information about how mothers (and daughters) can reduce breast cancer risk, we sought to increase women’s exposure to, satisfaction with, and acceptance of environmentally focused risk-reducing information. Thus, the first hypothesis (H1) was posited:

H1: The use of a targeted online blog intervention will increase blog reader’s exposure to and satisfaction with the breast cancer risk and prevention information compared with blog readers who are not exposed to (or who did not recall seeing) the intervention messages.

Given the interactive nature of blogging, we also wanted to encourage the diffusion of this information within women’s larger social networks. Previous research has not examined how intervention messages stemming from mommy blogs might influence interaction and information sharing among other social media platforms. Therefore, the following research question (RQ) was posed:

RQ1: How does the use of a targeted online blog intervention encourage interaction and information sharing about breast cancer risk and prevention messages across other online social media networks?

Behavior change is the ultimate goal of a health promotion intervention. According to the integrative model of behavioral prediction (a reasoned action theory approach to health promotion), several antecedents of behavior change should be evaluated to understand and predict whether women will take action [53,54]. These variables include acceptance of a health-related message, beliefs about health and risk, and intention to change. On the basis of this framework, we posited the second hypothesis (H2):

H2: The use of a targeted online blog intervention will increase breast cancer risk and prevention message acceptance, beliefs, and intentions to adopt the guidance.

Finally, to reach a larger audience, it is critical that this information is disseminated (or diffused) through channels perceived as optimal by women [15]. Therefore, we aimed to understand mothers’ preferred communication channels for breast cancer and environmental risk and prevention information. More than half of the US population use 2 or more social media platforms [55], and some individuals prefer traditional, interpersonal (eg, face-to-face) communication channels because of cultural norms about discussing health and risk topics [56]. To ensure women perceive mommy bloggers as an optimal channel but also provide opportunities to refine our intervention as necessary, we sought to learn about perceived optimal channels more broadly. Therefore, the following inquiry was posed:

RQ2: What are blog readers’ preferred media/communication channels for receiving information about breast cancer risk and prevention?

## Methods

This targeted social media intervention study involved a quasi-experimental design to assess women’s exposure to, acceptance of, and beliefs about environmental risks while promoting intentions to adopt risk-reducing behaviors.

### Participants and Recruitment

After the institutional review board approval, 3 groups of participants were recruited: (1) bloggers involved in the intervention, (2) readers exposed to the intervention (intervention group), and (3) readers not exposed to the intervention (comparison group). A convenience sample of mommy bloggers, all women, was recruited through The Motherhood [57], a network of more than 3000 diverse mothers who blog about various topics including health. During the recruitment process, efforts were made to find participants of different racial or ethnic groups and geographic locations, as well as prior experiences with breast cancer (either personally and/or by family members). A total of 75 mommy bloggers agreed to participate in the intervention (see Table 1 for blogger characteristics).

Upon consent, all participants completed an online survey with items about sociodemographics and breast cancer history. About 35 mommy bloggers had previously written about breast cancer in their blogs in the past year. All of the bloggers in the sample had children and 52 bloggers had daughters, specifically. Bloggers posted messages on their blogs to recruit readers for the intervention group.

The reader intervention group was comprised 445 blog readers (435 women and 10 men) who follow one or more of the 75 bloggers involved in the intervention. The participating mommy bloggers were asked to recruit their readers and direct them to an online postintervention survey link. A total of 353 blog readers (341 women and 12 men) made up the reader comparison group. To minimize contamination between the 2 groups, readers in the comparison group were recruited through a separate set of mommy bloggers who were affiliated with The Motherhood network, but who did not blog about breast cancer and were not involved in the intervention (see Table 2 for blog readers’ characteristics). The comparison group bloggers wrote about a wide range of issues, including parenting concerns and other health issues besides breast cancer.

**Table 1:** Sociodemographics and breast cancer or risk history of bloggers (n=75).

Characteristics		Bloggers
Age (years), mean (SD); range		37.88 (7.02); 25-61
Income (US $) based on reported zip code, median		65,611
**Race or ethnicity, n (%)**		
	White	43 (58)
	African American or black	15 (19)
	Asian or Pacific Islander	2 (3)
	Hispanic	14 (18)
	Other	1 (1)
**Education level, n (%)**		
	High school diploma	6 (8)
	Some college	13 (14)
	2-year college degree	6 (8)
	4-year college degree	29 (37)
	Graduate degree	21 (27)
**Breast cancer or risk history, n (%)**		
	Diagnosed with breast cancer	3 (4)
	Breast cancer 1 or 2 mutation positive	1 (1)
	Family history of breast cancer	50 (64)
	Diagnosed first-degree relative	13 (17)

**Table 2:** Sociodemographics and breast cancer or risk history of readers, intervention group (n=445) and comparison group (n=353).

Characteristics		Intervention group	Comparison group
Age (years), mean (SD); range		39.33 (10.82)^a^; 19-83	37.74 (10.18); 19-81
Average income (US $) by reported zip code		65,709	67,435
**Race or ethnicity, n (%)**			
	White	389 (87.4)	304 (86.1)
	African American or black	24 (5.4)	18 (5.1)
	Asian	13 (2.9)	5 (1.4)
	Hispanic	28 (6.3)	27 (7.6)
	Native American or Alaska native	5 (1.1)	4 (1.1)
	Other	11 (2.5)	3 (0.8)
**Education level, n (%)**			
	Less than high school	5 (1.1)	2 (0.5)
	High school graduate	37 (8.3)	26 (7.4)
	Some college	86 (19.3)	69 (19.5)
	2-year college degree	65 (14.6)	32 (9.1)
	4-year college degree	152 (34.2)	145 (41.1)
	Graduate degree	100 (22.4)	79 (22.4)
**Breast cancer or risk history, n (%)**			
	Diagnosed with breast cancer	16 (3.5)	10 (2.8)
	Breast cancer 1 or 2 mutation positive	7 (1.5)	7 (1.9)
	Family history of breast cancer	119 (26.7)	150 (42.4)
	Diagnosed first-degree relative	72 (16.2)	59 (16.7)

^a^Significant difference for age between intervention and comparison groups, *t*_794_=2.110; *P*=.03.

The reader intervention group completed an online postintervention survey after the message exposure period. In terms of frequency of use, 367 intervention group respondents reported reading mommy blogs one or more days per week (78 indicated less than once per week). Many (n=280) reported having daughters.

Blog readers recruited for the reader comparison group completed the same postintervention online survey as the intervention group. Respondents from this group (n=307) mentioned reading mommy blogs one or more days per week (46 reported less than once per week). Reader comparison group respondents (n=227) also reported having daughters. Participants in the intervention and comparison groups only differed significantly by age (*t*_794_=2.110; *P*=.03).

Intervention bloggers were provided incentives depending on their reach. Bloggers with an overall reach of up to 100,000 were given US $125; bloggers with an overall reach of up to 200,000 were given US $200; and bloggers with an overall reach above 250,000 were given US $300. Bloggers who disseminated the reader comparison group survey were offered US $50. Readers had the option to enter a raffle to win 1 out of 5 US $100 gift cards.

### Intervention Development and Dissemination

#### Message Development

**Figure 1:**
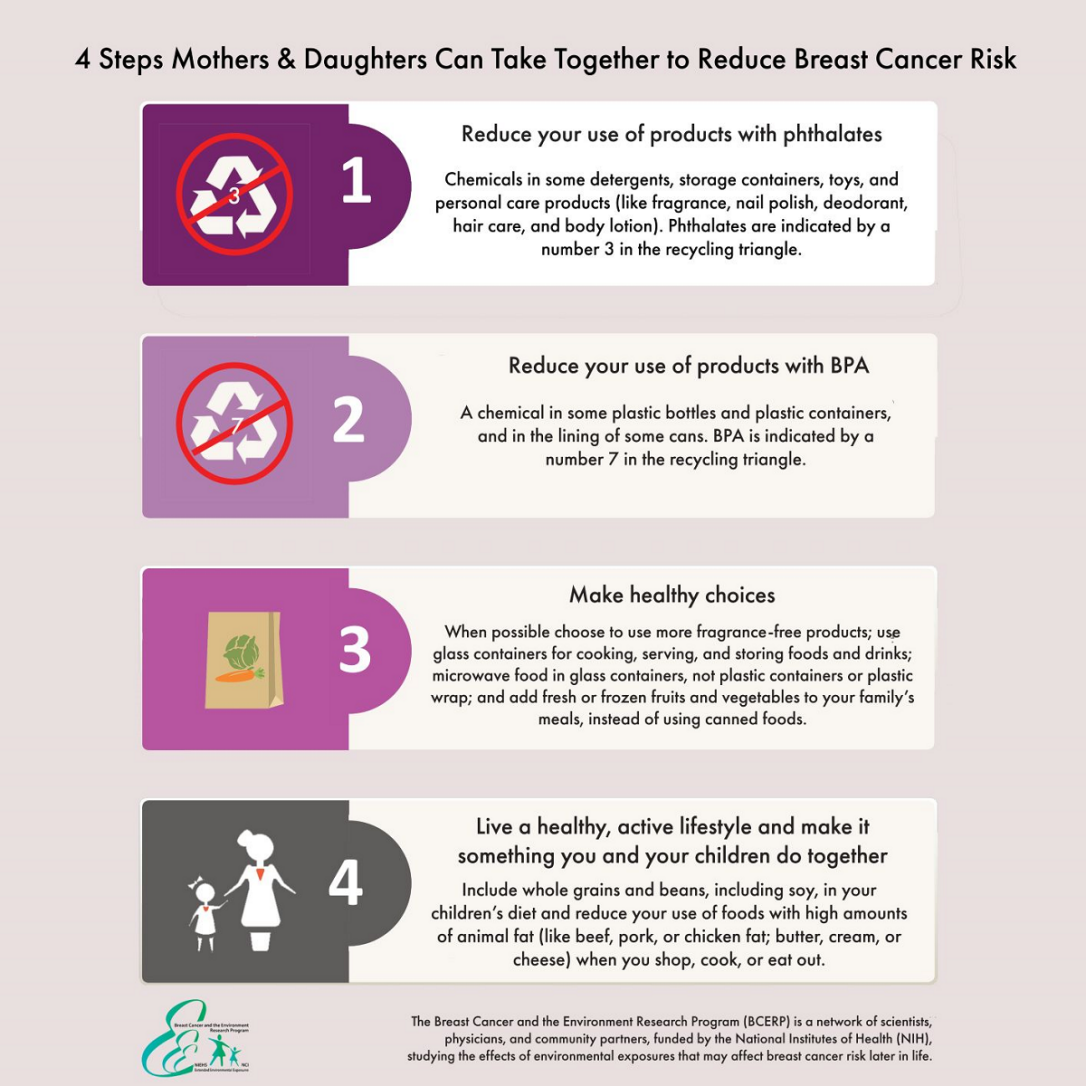
Intervention message adapted from the Breast Cancer and the Environment Research Program materials.

Our goal was to develop a message that was based on scientific evidence as well as informed by the community we sought to reach. First, to develop a scientifically informed message suitable for social media dissemination, 2 authors analyzed BCERP’s online educational materials targeting mothers and daughters [58]. The authors identified salient themes that would provide mothers with actionable steps they could take to reduce their own and their daughter’s environmental risk for breast cancer development. The remaining authors reviewed the analysis and confirmed the identification of 4 salient messages (or 4 steps mothers and daughters could take) communicated across the materials. To facilitate social media dissemination, a JPEG image with the 4 steps was created using language extracted from BCERP’s materials to ensure that information was accurate and uneditable (see Figure 1). Second, we sought to ensure that the message disseminated would also be community-informed (or user-generated) to better ensure women identified with the information. This aspect involved the bloggers adding their own content (which is further described below in procedures).

#### Intervention Procedures

Bloggers were provided with a document that included the study protocol and instructions about when and how to integrate the image or intervention message into their blog post. They also received procedures for completing the online survey and for recruiting their readers to do the same. Bloggers were asked to write a blog post during the National Breast Cancer Awareness Month (October-November 2017) that includes the image and targets mothers with daughters. In addition to the image, bloggers were asked to include 2 sentences of text to identify the source of the information (ie, BCERP). They were also given a link to additional online BCERP resources to include in their post.

**Figure 2:**
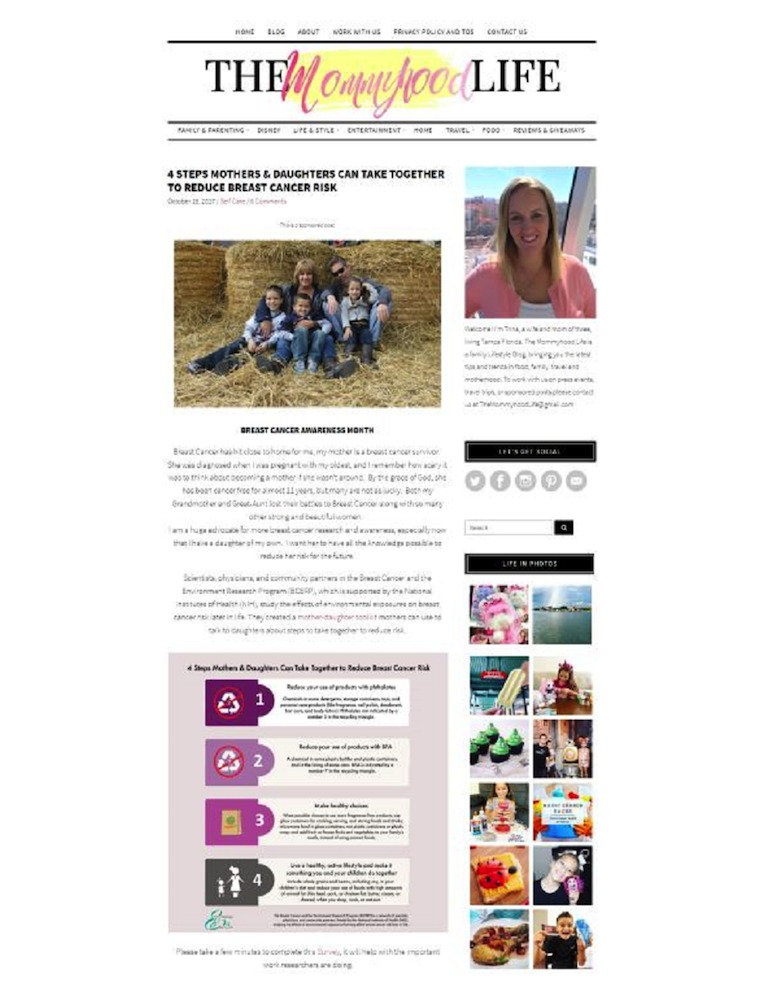
Example mommy blog post with adapted intervention message.

To ensure that information disseminated on the blog was in part user-generated [15], bloggers were asked to use their experience with their blog audience to integrate the image and required text in a manner that they felt would be appealing to them (see Figure 2). Bloggers were not required but encouraged to promote their post via other social media channels (eg, Facebook, Twitter, and Instagram) and to consider using the hashtags #BCERP, #MotherDaughter, #BreastCancerRisk, and/or #BreastCancerAwareness in their posts. They were told that they could provide a link to their original blog post or link to the BCERP toolkit directly. Finally, bloggers were asked to post a message with links to the surveys to remind readers to complete the online reader survey.

#### Analysis of Breast Cancer Messages Associated With Seeded Mommy Blogs

To assess the degree to which the intervention encouraged interaction about breast cancer risk messages among blog users (research question [RQ] 1), the researchers used an online tracking program, *Tracx* [59], to capture blog and related online social media content associated with the targeted online blog intervention. *Tracx* is a social media monitoring and analytics program that uses Boolean search querying to track posts, mentions, and conversations across different online channels (eg, social media platforms, news outlets, blogs, and forums) through the program algorithms. Additionally, it populates the number of author’s followers and total engagements per post.

The sample was collected between October 1 and December 31, 2017, by one of the authors, corresponding with the start of the intervention and 2 months following it. To ensure exhaustiveness, manual identification of posts and ensuing reactions across all platforms was performed in addition to using *Tracx.* Out of 7897 total posts reported, 293 additional blog posts, social media posts, and engagements were added manually.

The frequency of the following key variables was reported by *Tracx*: A post was the initial piece of breast cancer content submitted to a blog post and/or other social media used by the participating bloggers to reach their reader audience. Engagement referred to any interaction with a post on a social network including Google+, Facebook, Twitter, and Instagram. An interaction included likes, retweets, replies, shares, favorites, and comments in relation to the original post. Comments were defined as any text or emoji response to a post on either the original blog post or a post on social media.

**Figure 3:**
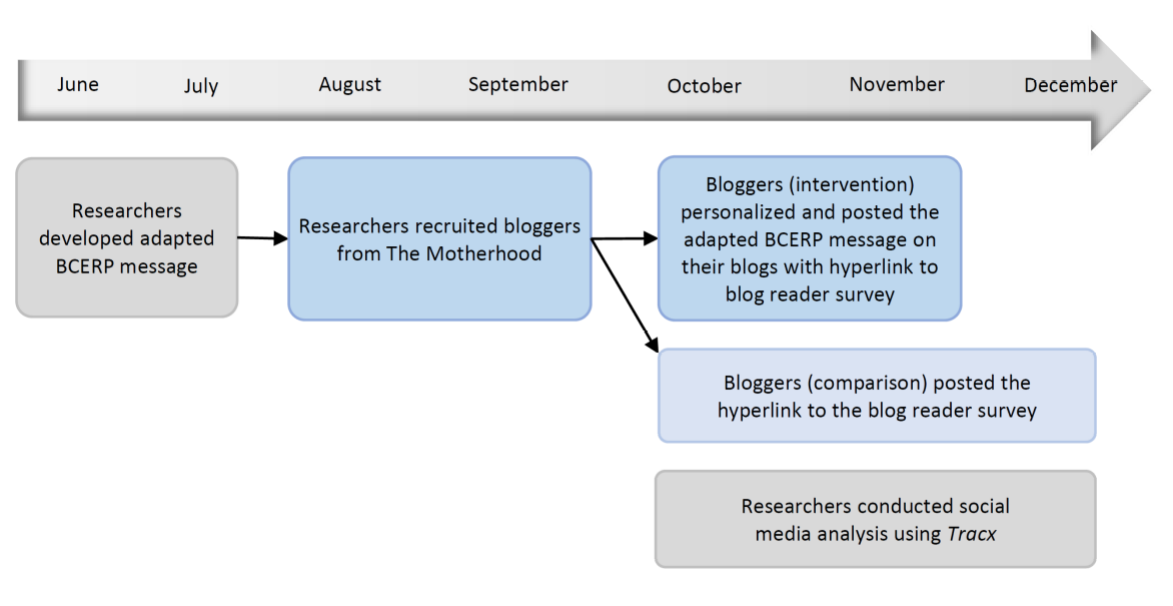
Intervention process and timeline (2017). BCERP: Breast Cancer and the Environment Research Program.

Reach was operationalized as the number of unique people who were potentially exposed to the original blog posts. The overall reach of bloggers was calculated by the number of readers who followed their blog. Multiple reactions from the same person across channels were counted as one reach. *Tracx* was able to match up the engagement, comment, and reach with each mommy blogger original post. Figure 3 demonstrates the flow and timeline of the intervention process.

### Blog Reader Survey Measures

The blog reader survey included a variety of measures that are consistent with the diffusion of innovation theory [14], including measures of message exposure, message satisfaction, message acceptance or influence, and likelihood of sharing the BCERP messages with the members of other social networks.

#### Intervention Message Recall

Recall of the intervention messages was assessed by the following question: *Did you notice breast cancer risk and prevention information from the BCERP (Breast Cancer and the Environment Research Program of the National Institutes of Health) organization in any of the blog posts you read recently?* Respondents were also asked to list up to 5 different blogs they followed in the past year to help the researchers detect exposure to the intervention message on other blogs the readers follow. In this way, the researchers could also determine whether reader comparison group participants had been exposed to the intervention message.

#### Exposure to Breast Cancer Risk and Prevention Information

Exposure to information about breast cancer risk and prevention was measured with a 5-point Likert-type scale item: *The blog(s) I follow has/have increased my exposure to information about breast cancer risk and prevention*, ranging from *Strongly Disagree* to *Strongly Agree*.

#### Satisfaction With Breast Cancer Risk and Prevention Information From Blogs

Satisfaction with the breast cancer risk and prevention information that blog readers were exposed to was measured using a 5-point Likert-type scale item: *Please indicate how satisfied you are with the way the blog(s) you follow present breast cancer risk and prevention information*, ranging from *Not Satisfied at All* to *Very Satisfied*.

#### Breast Cancer Risk and Prevention Message Acceptance or Influence

Breast cancer risk and prevention message acceptance or influence was measured with 3 5-point Likert-type scale items, including: *How much did reading the recent breast cancer risk and prevention blog posts influence your acceptance of the message regarding breast cancer risk?*; *How much did reading the recent breast cancer risk and prevention blog posts influence your beliefs about breast cancer risk?*; and *How much did reading the recent breast cancer risk and prevention blog posts influence your behaviors to reduce your risk of breast cancer?* Each response ranged from *Not at All* to *Very Much*. These measures were added together to create a composite score (Cronbach alpha=.87).

#### Likelihood of Sharing Breast Cancer Risk and Prevention Information From Blogs

Participant likelihood of sharing breast cancer risk and prevention information from blogs was measured with a 5-point Likert-type scale item: *In the future, how likely are you to share with others any information about breast cancer risk and prevention from the breast cancer risk blog post(s) you read in the past year?*, ranging from *Not Likely at All* to *Very Likely*.

#### Sharing of Breast Cancer Risk and Prevention Information

All participants were asked, *With whom did you share the information from the breast cancer risk and prevention blog post(s) you read?* Participants could make their selection from a drop-down menu that included various family members, friends, coworkers, and others within their social networks.

#### Perceived Importance of Breast Cancer Risk and Prevention as a Topic

Participant perceptions of the importance of breast cancer risk and prevention as a blog topic was measured with a 5-point Likert-type scale item: *How important to you is breast cancer risk and prevention as a topic?*, ranging from *Unimportant* to *Very Important*.

### Statistical Analyses

#### Significance Tests

Statistical analyses were conducted using IBM SPSS. Independent sample *t* tests were performed in an initial assessment of differences between blog reader intervention and comparison groups. Analysis of covariance (ANCOVA) was conducted to test H1 and H2. Finally, a chi-square analysis was performed to analyze between-group differences in sharing breast cancer risk and prevention information with others, specifically daughters. Values were considered statistically significant at *P*<.05.

#### Initial Comparison of Blog Reader Intervention and Comparison Groups

In terms of the perceived importance of breast cancer risk and prevention as a topic, there was no significant difference between members of the intervention and comparison groups, *t*_794_=−1.004; *P*=.31. As expected, participants in the reader intervention group were significantly more likely to recall seeing one or more of the intervention messages (n=165), χ^2^_1_=42.9; *P*<.001. However, 32 individuals in the comparison group also recalled seeing the intervention messages on 1 of 5 other blogs they follow. These individuals were treated as members of the intervention or exposed condition in analyses, bringing the total number of participants who recalled seeing one or more of the intervention messages to 197 (n=197) versus the comparison group (n=321).

## Results

H1 posited that the use of a targeted online blog intervention would increase blog reader’s exposure to and satisfaction with the breast cancer risk and prevention information they received compared with blog readers who were not exposed to (or who did not recall seeing) the intervention messages. An ANCOVA test comparing breast cancer risk and prevention information exposure scores between participants who recalled seeing the BCERP intervention messages versus those who did not, while controlling for level of education, revealed that participants who recalled seeing the intervention messages had higher breast cancer risk and prevention information exposure scores (mean 3.92, SD 0.85) than those who did not (mean 3.45, SD 0.92; *F*_1,409_=25.78; *P*<.001; partial eta^2^=0.06). In addition, those readers who recalled the intervention messages had higher breast cancer risk and prevention information satisfaction scores (mean 3.97, SD 0.75) than participants who did not recall seeing the intervention messages (mean 3.57, SD 0.94; *F*_1,409_=19.86; *P*<.001; partial eta^2^=0.047) supporting H1.

RQ1 asked how a targeted online blog intervention might encourage interaction or information sharing about breast cancer risk and prevention messages across other social media networks. The *Tracx*-assisted content analysis of the breast cancer messages associated with the participating bloggers’ adapted intervention messages captured message reach, message engagement, and number of comments as detailed in Table 3.

**Table 3:** Frequencies of message posts, reach, engagement, and comments.

Key metrics for social networking sites, n	Blog	Google+	Facebook	Twitter	Instagram	Total
Posts	75	12	25	98	12	222
Reach^a^	310	21	247	648	1048	2153
Engagement^b^	1778	11	432	1742	3712	7675
Comments	283	0	60	0	181	524

^a^Defined as the number of unique people who were potentially exposed to the original blog posts.

^b^Defined as any interaction with a post on a social network.

Moreover, a chi-square test revealed that blog readers who recalled seeing the intervention messages were significantly more likely to share the breast cancer risk and prevention information they read, with their daughters specifically, when compared with individuals who did not recall seeing them, χ^2^_1_=8.1; *P*=.004.

H2 predicted that a targeted online blog intervention will influence breast cancer risk and prevention message acceptance, beliefs, and intentions to act on the information provided to reduce breast cancer risk. An ANCOVA test comparing breast cancer risk information influence scores of intervention and comparison groups, while controlling for level of education (*F*_1,407_=13.89; *P*<.001) and age (*F*_1,407_=8.93; *P*=.003), showed significantly higher breast cancer risk and prevention information influence scores (mean 11.22, SD 2.93) for those who recalled seeing the intervention message when compared with those who did not (mean 10.14, SD 3.24; *F*_1,407_=9.16; *P*=.003; partial eta^2^=0.022, supporting H2).

The second research question (RQ2) asked about mommy blog readers’ media or channel preferences for receiving future information about breast cancer risk and prevention. For the intervention group, 14.4% (64/445) of participants ranked Facebook as their first choice for receiving future information about breast cancer risk, followed by 0.07% (31/445) who ranked Twitter as their first choice, 0.05% (22/445) who ranked *other social media* first, 0.02% (8/445) who ranked blogs as their first choice, and 0.01% (4/445) who ranked email as their first choice.

In the comparison group, 0.09% (35/353) of participants ranked Facebook as their first choice for receiving future information about breast cancer risk and prevention, 0.06% (21/353) ranked Twitter as their first choice, 0.01% (6/353) ranked *other social media* as their first choice, 0.01% (5/353) ranked blogs as their first choice, 0.008% (3/353) ranked email as their first choice, and 0.003% (1/353) ranked mail (eg, postal system) as their first choice. In terms of other preferred social media, the most frequently mentioned channels were Instagram (n=27) and Pinterest (n=9). Other suggested channels for disseminating breast cancer risk and prevention messages were YouTube (n=3), text messages (n=3), medical websites, such as WebMD or PubMed (n=2), Google (n=2), and Tumblr (n=1).

## Discussion

### Principal Findings

The aim of this study was to examine whether a targeted intervention in which mommy bloggers disseminate evidence-based information could increase mothers’ exposure to and dissemination of breast cancer environmental risk and prevention information and, ultimately, be a potential means of persuading mothers to engage in behavioral changes that could reduce their disease risk. To better determine the potential for women to engage in risk-reducing lifestyle behaviors advocated for in the messages, we investigated several antecedents to behavior change (message satisfaction, acceptance and beliefs about the information, and behavioral intentions to adopt the guidance). Additionally, to ascertain the potential reach of this intervention approach, we explored women’s dissemination of and preferences for information via other social media platforms, allowing us to explore the potential for rapid diffusion via this intervention approach.

Results indicated that mommy blog readers who were exposed to (and specifically recalled) the seeded intervention message adapted from BCERP guidance had higher breast cancer risk information exposure scores and higher breast cancer risk and prevention information satisfaction and influence scores than those who did not see (or recall) them. These findings are consistent with previous theory and research on the influence of social networks and opinion leaders within them on health perceptions [14,19,32,45]. Thus, mommy bloggers may serve as important opinion leaders for some women. Moreover, mommy bloggers may be key to enhancing the messaging, delivery, and impact of environmental breast cancer risk information on mothers.

Our intervention involved both evidence-based and user-generated message development. As such, our targeted intervention demonstrated that future designers of breast cancer communication interventions can provide online opinion leaders such as mommy bloggers with health information adapted from evidence-based research from authoritative groups (eg, National Institutes of Health [NIH]). This may be especially important in better ensuring that information posted on blogs is accurate and credible given a recent study that showed that blogs are often less accurate than other online sources [12]. Our findings also show that it is important to include content generated by the user (blogger). Online opinion leaders can tailor more stylistic aspects of intervention messages and communicate them according to their readers’ needs, experiences, and preferences. Using established relationships between bloggers and their readers may provide a more organic and collaborative means of tailoring health-related messages compared with interventions where the message tailoring is conducted by the researchers alone (based on target audience member feedback and characteristics). Future studies should compare similar types of message-seeding strategies via established opinion leaders with more traditional message tailoring approaches to further assess their potential variant influence on health outcomes.

In addition, findings revealed that disseminating health messages through opinion leaders may influence the further dissemination and amplification of those messages, meaning that there is potential for rapid diffusion of information via social media interventions. Findings of the *Tracx* analysis indicated that initial mommy blog message posts were shared on a variety of social networking sites, including Facebook, Twitter, Instagram, and other blogs, with Instagram being the most widely used. However, data from the blog reader survey (RQ2) indicated that participants varied widely in terms of self-reported preferences for how they would like to receive environmental breast cancer risk information in the future. Overall, these findings suggest that disseminating health information through blogs may have an extensive reach. Information may be encountered by individuals in one social media platform and then shared with others across other (perhaps more preferred) social media. Our *Tracx* data on message reach showed that some form of the initial seeded intervention messages from the participating bloggers was shared well beyond their initial blog reader audience.

Moreover, this study found that reach, or rapid diffusion of information, extended beyond online communities and platforms to individuals’ personal networks. Our data showed that mommy bloggers’ posts spurred interaction about breast cancer risk among women and their daughters, specifically. This finding is an important reminder that health information encountered via social media is often shared in close relationships, which may reinforce the impact of the information on health behaviors. This finding is consistent with Rogers’ diffusion of innovation theory [14] and associated research that illustrates a 2-step flow of information from media to interpersonal conversations. Future research would benefit from the use of social network analysis to explore the spread of message dissemination among bloggers, their readers, and other online or face-to-face social network members.

The findings also prompt questions about how to best prepare women who use blogs and other social media for conversations with their daughters about environmental breast cancer risk. Communication competence is particularly of concern when mothers talk to their daughters about risk-related topics since these conversations can increase psychological and physiological distress for daughters [3,50]. Other factors, including culture, relational history, and age or maturity, make these conversations even more complex to navigate. Future studies should consider messages that incorporate tailored guidance for engaging in family conversations about breast cancer risk.

Moreover, the findings indicated that the intervention message had a modest impact on key study measures of exposure, satisfaction with the message, intentions to act on the information, and likelihood of sharing the information with others. One intervening variable was participant’s recall of the message. Although we know that individuals who recalled the message were more exposed and satisfied with them, and they had stronger intentions to act on the information than the comparison group, it is unknown whether they were recalled from the original blogger post, comments from online or face-to-face social network members, or another social networking site (eg, Facebook and Instagram). Future researchers should take this into account when attempting similar types of seeded messaging strategies. Although researchers can track where information has been reposted or replied to using programs such as *Tracx*, it is often difficult to assess (outside of self-reports) where people encountered specific intervention messages within the social media landscape or how these transfer to interpersonal conversations. In addition, since 42% of women in the comparison group had a family history of breast cancer, it is likely that these individuals had a keen interest in breast cancer prevention and could have been prone to seek this information on blogs (increasing the chance for contamination between the treatment and comparison groups).

The findings also suggest that Instagram was the most popular *other* social media platform for blog readers. This is likely because of Instagram’s ability to conveniently share or obtain breast cancer information on a variety of other social media channels (such as Facebook and Twitter). Future research should examine how Instagram (and similar platforms) may enhance the reach and impact of future interventions that attempt to seed similar online opinion leaders with evidence-based breast cancer and environmental risk and prevention messages.

### Limitations

There are a number of limitations in this study. First, the study relied on a convenience sample of participants using a quasi-experimental design as opposed to a randomized treatment or control group study. One of the reasons for this was the desire to work with naturally occurring bloggers and their established online social networks of readers. However, the lack of randomized groups limits the generalizability of claims regarding the efficacy of the seeded mommy blogger intervention used in this study.

Additionally, the bloggers and blog readers tended to be highly educated overall. Less than 10% of the intervention group or comparison group members had a high school education or less. The researchers are in the process of analyzing qualitative interview data from a subset of ethnic minority and lower socioeconomic status respondents who participated in this study to assess how intervention messages could be tailored in more culturally sensitive and educationally appropriate ways in future research.

Yet, the findings provide insights into the value of this approach while highlighting consideration for future research. One future direction would be to systematically assess what factors are associated with greater likelihood of recall among participants in this type of intervention. The education level was controlled in this study, but other factors associated with education, such as health (or electronic health) literacy, could influence message recall. To explore the impact of such factors on participant recall, studies might employ interviews and/or other qualitative methods to see how user-centered design can be integrated with scientifically accurate information to develop more memorable messages.

In terms of other limitations, blog readers typically follow more than one blog, and this might have led to contamination between the intervention and comparison groups. However, we attempted to correct this by asking readers to report up to 5 blogs they visited most frequently in the past year. This allowed us to discover individuals in the comparison group who saw the intervention message. Furthermore, we were limited by the number of survey items we could reasonably include, which contributed to not being able to use larger multidimensional measures of the key variables of interest. In future research, we hope to capture more nuanced aspects of complex variables, such as behavioral intentions to act on environmental breast cancer guidance, as well as finding ways to better distinguish between people who actively disseminate intervention messages and those who are more passive recipients of these messages (ie, lurkers).

Finally, the use of longitudinal research designs is important when assessing health knowledge and behavior change that may result from a social media intervention such as the one reported here. Given that the reader survey was administered over a 2-month period following the start of the mommy blogger intervention, it is difficult to know about the long-term effects of exposure to these types of messages. Future studies need to find creative ways to measure variables longitudinally in an environment that is dynamic in terms of online network membership and the stability of specific social media channels (eg, social media platforms may decline in popularity over time and users may shift to newer platforms). This presents numerous challenges in terms of deciding the best channel(s) for health intervention messages, including those regarding environmental breast cancer risk. Moreover, future researchers should consider assessing mommy bloggers’ satisfaction regarding their participation in terms of serving as opinion leaders in similar interventions.

### Conclusions

The study results showed promise for the utilization of online opinion leaders such as mommy bloggers as influential channels for the dissemination of both evidence-based and user-generated environmental breast cancer risk messages. Moreover, this study shed light on how blog readers share environmental breast cancer risk and prevention messages across different social media platforms and the preferred channels for receiving this type of information. Results also revealed the potential reach of disseminating health risk information via bloggers. Mommy bloggers may be especially primed to disseminate health risk information geared toward mothers, and this channel may effectively serve as a catalyst for mother-daughter communication about breast cancer risk reduction. Mommy bloggers may also aid interventionists in enhancing the messaging, delivery, and impact of environmental breast cancer risk information.

## Abbreviations

ANCOVA: analysis of covariance
BCERP: Breast Cancer and the Environment Research Program
BPA: bisphenol A
H1: first hypothesis
H2: second hypothesis
IBCERCC: Interagency Breast Cancer and Environmental Research Coordinating Committee
NCI: National Cancer Institute
NIEHS: National Institute of Environmental Health Sciences
NIH: National Institutes of Health
RQ: research question
