# Metadata Correction: Partnering With Mommy Bloggers to Disseminate Breast Cancer Risk Information: Social Media Intervention

**DOI:** 10.2196/14158

**Published:** 2019-06-17

**Authors:** Kevin Wright, Carla Fisher, Camella Rising, Amelia Burke-Garcia, Dasha Afanaseva, Xiaomei Cai

**Affiliations:** 1 George Mason University Fairfax, VA United States; 2 University of Florida UF Health Cancer Center Gainesville, FL United States; 3 Westat Rockville, MD United States

The authors of “Partnering With Mommy Bloggers to Disseminate Breast Cancer Risk Information: Social Media Intervention” (J Med Internet Res 2019;21(3):e12441) made an error when listing the affiliation of author Carla Fisher.

Her affiliation was previously “College of Health and Human Performance, University of Florida, Gainesville, FL, United States” and has now been changed to “University of Florida, UF Health Cancer Center, Gainesville, FL, United States”.

The correction will appear in the online version of the paper on the JMIR website on June 17, 2019, together with the publication of this correction notice. Because this was made after submission to PubMed, PubMed Central, and other full-text repositories, the corrected article also has been resubmitted to those repositories.

